# Utility of the Clinical Assessment Scale for Autoimmune Encephalitis (CASE) Score to Define Relapse in the Scarcity of Biomarker Footprints in Anti-Leucine-Rich Glioma-Inactivated Protein 1 Encephalitis: A Case Report

**DOI:** 10.7759/cureus.93405

**Published:** 2025-09-28

**Authors:** Saeko Adachi, Tatsuki Matsuda, Naomi Kanazawa, Takahiro Iizuka, Kensuke Shiga

**Affiliations:** 1 Department of Neurology, Matsushita Memorial Hospital, Moriguchi, JPN; 2 Department of Neurology, Kobe Central Hospital, Kobe, JPN; 3 Department of Neurology, Kitasato University School of Medicine, Sagamihara, JPN

**Keywords:** antibody titer level, autoimmune encephalitis, cell-based assay, clinical score, lgi1 antibody

## Abstract

Anti-leucine-rich glioma-inactivated protein 1 (anti-LGI1) encephalitis is an autoimmune encephalitis caused by autoantibodies against LGI1 and often presents with subacute cognitive decline, behavioral symptoms, and seizures. Relapses can occur after a favorable treatment response to the first-line immunotherapy, whereas decision-making on relapses may sometimes be challenging. Here, we report the case of a 71-year-old woman who developed cognitive decline, psychiatric symptoms, and faciobrachial dystonic seizures over three months. The patient was diagnosed with anti-LGI1 encephalitis based on high signal intensity on fluid-attenuated inversion recovery in the medial temporal lobes and antibody test results. One month after improvement with first-line immunotherapy, psychiatric symptoms and cognitive decline relapsed. The neurological findings and anti-LGI1 antibody profiles at the relapse did not converge on the typical constellation of diagnostic signatures of anti-LGI1 encephalitis. However, the deterioration of the Clinical Assessment Scale for Autoimmune Encephalitis (CASE) score indicated substantial worsening of symptoms. The case highlights that the assessment using the CASE score over time may be beneficial to define relapse in a setting with insufficient laboratory evidence of anti-LGI1 encephalitis.

## Introduction

Leucine-rich glioma-inactivated protein 1 (LGI1) constitutes a voltage-gated potassium channel complex present in the presynaptic membrane and is involved in the development and physiology of the central nervous system. Anti-LGI1 encephalitis is an autoimmune encephalitis caused by autoantibodies against LGI1 [1]. Neurological symptoms encompass subacute cognitive decline, a variety of behavioral or psychiatric symptoms, and seizures, including pathognomic faciobrachial dystonic seizures (FBDS). The laboratory hallmarks comprise hyponatremia, positive LGI1 antibodies in serum and cerebrospinal fluid (CSF) using cell-based assay (CBA), tissue-based assay (TBA) using rat hippocampus and cerebellum [2], and increased signals on fluid-attenuated inversion recovery (FLAIR) highly restricted to the medial temporal lobes [3]. Whereas neurological symptoms improve by first-line immunotherapy in approximately 80% of patients [3], clinical relapse can occur in 16.2-35% [3-5].

However, decision-making on the recurrence in patients with anti-LGI1 encephalitis remains a diagnostic conundrum, especially in patients with substantial cognitive sequelae. First, because it is sometimes difficult to distinguish a relapse of psychiatric symptoms from a fluctuation of residual behavioral or psychiatric symptoms. Second, because diagnostic biomarkers in anti-LGI1 encephalitis, such as increased antibody titers or apparent worsening of the medial temporal lobe MRI lesions, are sometimes absent in relapse [5].

The Clinical Assessment Scale for Autoimmune Encephalitis (CASE) score was developed as a measure of the severity of autoimmune encephalitis, with high scores reflecting severe disease activity [6]. Using the CASE score as a parameter of clinical deterioration in a setting of relapse can be helpful in patients with negligible or disputable radiological or serological hallmarks. Here, we present the case of a patient with anti-LGI1 encephalitis, in which the CASE score was beneficial in making clinical judgment of relapse amidst the scarcity of typical biomarkers of anti-LGI1 encephalitis.

## Case presentation

A 71-year-old woman was referred to our ambulatory department due to cognitive decline and refractory seizures over three months. The past medical history included hypertension and an ischemic stroke in the right anterior cerebral artery territory 10 months before the referral. As a neurological sequel of the stroke, she had a mild left hemiparesis, predominantly affecting the lower extremity. Until three months before the referral, she was able to ambulate with a cane. Over the three months, her level of activities of daily living gradually deteriorated to a point where she was wheelchair-bound at the time of her presentation. Furthermore, three months before the visit, she started to develop recurrent seizures in the left face and extremities, which were initially diagnosed as post-stroke epilepsy by a referring physician. Typical seizures consisted of a short spasm over the left face and the left upper extremity with dystonic posture, usually lasting for seconds to a minute. Her spouse reported that the spasms occurred about once an hour at the time of her visit to our hospital. She was treated with lacosamide at a dose of 200 mg per day, then switched to perampanel at a daily dose of 4 mg, both of which resulted in poor treatment response to seizures. Two months before this referral, she started to manifest memory impairment, somnolence, and delusional symptoms, including visual hallucinations. One month before the visit, she became less talkative and started to have difficulty eating on her own. She was able to walk only a few meters with substantial physical assistance.

On neurological examination at the ambulatory service, she rarely spoke and made only occasional eye contact with a physician. She could not follow the gaze with smooth pursuit eye tracking. The face was symmetric, and the Barré sign was negative. She did not exhibit bradykinesia. Muscle tone was moderately increased with paratonic rigidity in all extremities. Reflexes were brisk and symmetric without Babinski signs. The neck was supple. During the examination, she happened to present a dystonic seizure lasting a few seconds on the left side of her face and the left upper extremity, which was considered FBDS. The CASE score was 22.

**Figure 1:**
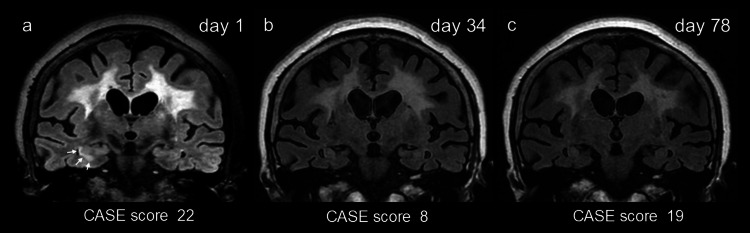
Chronological changes on brain MRI and Clinical Assessment Scale for Autoimmune Encephalitis (CASE) score. Note increased signal on fluid-attenuated inversion recovery (FLAIR) in the right hippocampus on the day of admission (a, arrows), but no apparent high signal on the follow-up MRIs obtained after recovery of symptoms (b) and even on the day of readmission due to clinical relapse (c). Panels a, b, and c were obtained on the day of admission (day one), after two cycles of intravenous high-dose methylprednisolone and four rounds of plasma exchange (day 34), and on the day of readmission due to clinical relapse (day 78), respectively. Panel (a) was acquired with a 3 Tesla scanner, while panels (b) and (c) were performed on a 1.5 Tesla scanner. The diffuse FLAIR high intensity in the white matter was considered as pre-existing ischemic changes because this finding was already seen on the MRI scans taken 10 months before the presentation to our facility (data not shown).

The serum sodium was 129 mmol/L (normal range: 138-145 mmol/L). CSF analysis was as follows: cell count: 1/µL (normal range: 0-5/µL), protein: 39 mg/dL (normal range: 15-45 mg/dL), and IgG index: 0.41 (normal range: <0.7). CSF-restricted oligoclonal bands were negative. Brain MRI showed increased fluid-attenuated inversion recovery (FLAIR) signal in the right hippocampal CA1 region (Figure 1, Panel a).

**Figure 2:**
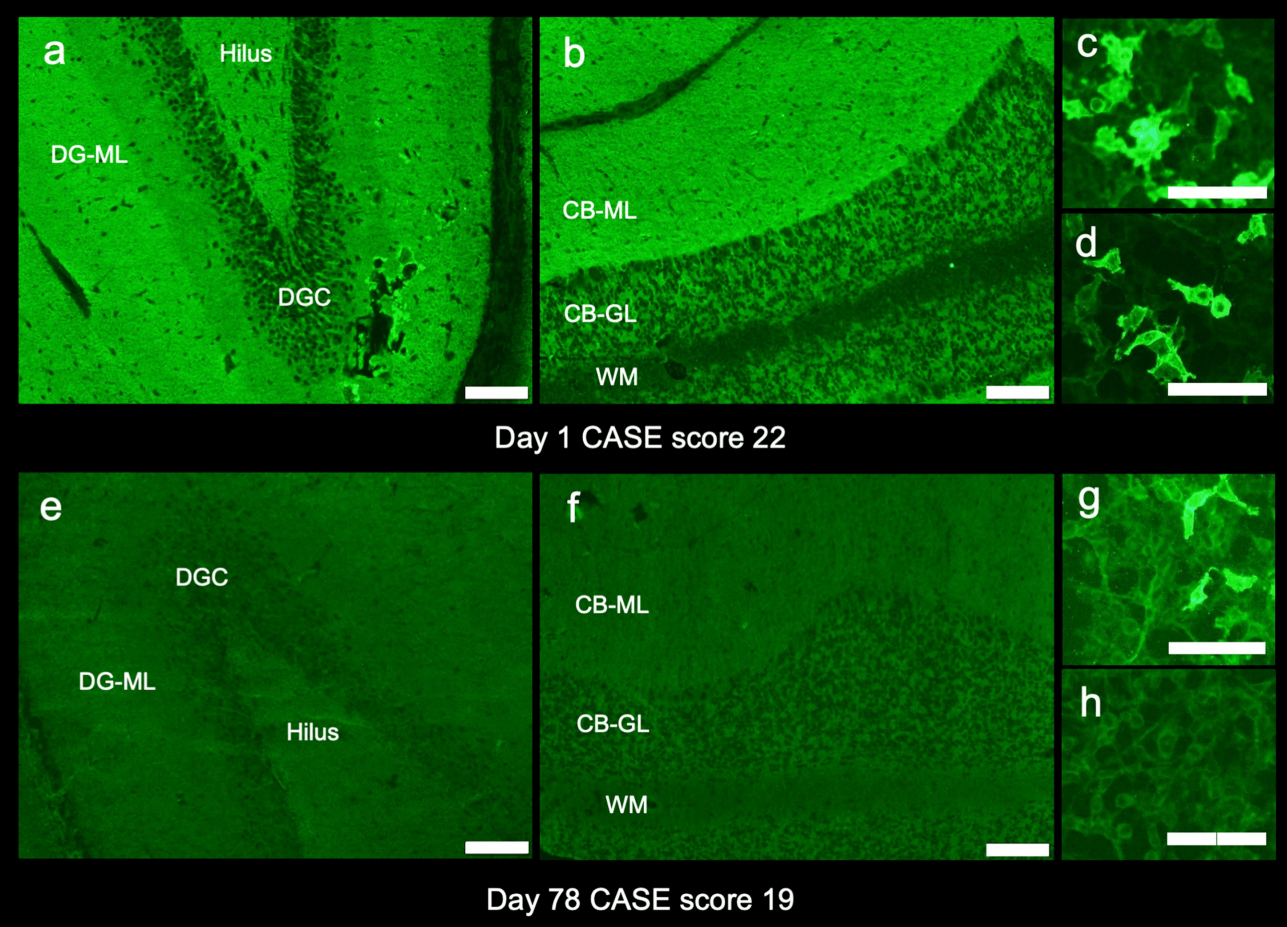
Antibody assays. Antibody assays were performed with indirect immunofluorescence tissue-based assay (TBA) and fixed cell-based assay (CBA) using cerebrospinal fluid (CSF) (1:2 dilution) and serum (1:20 dilution) obtained on the day of admission (day one, a-d) and readmission due to clinical relapse (day 78, e-h). Note intense neuropil reactivity in the middle layer of the dentate gyrus molecular layer (a) and in the cerebellar molecular layer (b), consistent with leucine-rich glioma-inactivated protein 1 (LGI1) reactivity [2]. The presence of LGI antibodies was confirmed in both serum (c) and CSF (d) by fixed CBA. Follow-up studies at relapse of symptoms reveal no apparent neuropil reactivity on either hippocampus (e) or cerebellum (f). Follow-up CBA shows mild reactivity in the serum (g) but not in the CSF (h). Panels a-d were performed on the day of admission (day one), and panels e-h were performed on the day of readmission (day 78) (scale bar = 100 µm). CB-GL: cerebellar granular cell layer; CB-ML: cerebellar molecular layer; DGC: dentate granule cells; DG-ML: dentate gyrus molecular layer; WM: cerebellar white matter

First, the patient’s CSF (1:2 dilution) was screened at Kitasato University with a commercial kit (EUROIMMUN AG, Lübeck, Germany) using indirect immunofluorescence (IIF) assays. This kit consists of four biochips per field, containing the N-methyl-D-aspartate receptor-transfected cells, control-transfected cells, hippocampus, and cerebellum. Based on the neuropil pattern on IIF-TBA consistent with LGI1 reactivity (Figure 2, Panels a, b) [2], we additionally performed fixed CBA expressing LGI1, and Casper2 (EUROIMMUN AG, Lübeck, Germany) and confirmed the presence of LGI1 antibodies in both serum (1:10 dilution) and CSF (1:2 dilution) (Figure 2, Panels c, d). A variety of neuronal surface (NS) antibodies were further investigated at the laboratory of Josep Dalmau (Dalmau’s Lab, Barcelona) with established assays (in-house rat brain immunohistochemistry (IHC) adapted to NS antigens and in-house CBA). IHC revealed a reactivity compatible with LGI1 antibodies but not reactivity suggesting the presence of concurrent other NS antibodies. The LGI1 antibodies were confirmed by in-house live CBA.

The diagnosis of LGI1 encephalitis was made based on typical clinical features with detection of LGI1 antibodies on two independent assays. On the day of admission, intravenous high-dose methylprednisolone (IVMP, 1,000 mg/day, three days) was administered. The recurrent episodes of FBDS disappeared soon after the initiation of IVMP without modifying the regimen of anti-seizure medication. Six days after IVMP, four rounds of plasma exchanges (PEs) were performed. After the treatment with IVMP and PE, her arousal level improved, and she became able to eat on her own. To further improve her symptoms, the second IVMP was administered immediately after the completion of PE. After the second IVMP, she was able to talk more words in daily conversation and was able to walk with slight assistance. Oral prednisolone was started at 30 mg per day after the second IVMP, then gradually tapered to 10 mg per day. Her cognitive impairment remained to a certain extent. The increased FLAIR signals in the right hippocampus disappeared on day 34 (Figure 1, Panel b). She was transferred to a nursing facility on oral prednisolone of 10 mg per day, with a CASE score of 8.

One month later, somnolence and restlessness developed at the facility, and she was readmitted to our hospital. On admission, she was able to talk to some extent, but her vocabulary was imprecise and incoherent. She was not able to open both eyes with some dystonic features, even though she seemed to understand that she was told to open them by a physician. Seizures, including FBDS, had not been observed since she was discharged from our hospital. The CASE score was 19.

On admission (78 days after the first admission), the serum sodium was 143 mmol/L. CSF analysis was as follows: cell count: 0/µL, protein: 46 mg/dL, and IgG index: 0.64. No increased signal was seen in the medial temporal lobe on either T2/FLAIR or diffusion-weighted imaging (Figure 1, Panel c), while substantial atrophy was noticed in the hippocampus bilaterally (Figure 1, Panel c). IIF-TBA using CSF did not reveal apparently increased reactivity in either dentate gyrus molecular layer or cerebellar molecular layer (Figure 2, Panels e, f). Although the intensity of the reactivity decreased, LGI1 antibodies remained positive in serum but negative in CSF (Figure 2, Panels g, h). These test results suggested a reduction in LGI1 antibody titers in the follow-up samples; however, a possible relapse of encephalitis was suspected based on psychobehavioral deterioration, as evidenced by the 11-point increase in the CASE score.

Following two additional cycles of IVMP (1,000 mg/day, three days), her psychiatric symptoms ameliorated, with a CASE score of 11. Additional plasma exchange or second-line immunotherapy was not added because of the preference of the patient’s family. She was then transferred to the nursing facility with prednisolone 10 mg per day. The CASE score slightly improved to 8 in three months after the second discharge. The specific items of these CASE scores are shown in Table 1.

**Table 1:** The Clinical Assessment Scale for Autoimmune Encephalitis (CASE) score.

Items	Day 1	Day 34	Day 78	Day 241
Seizure	3	0	0	0
Memory dysfunction	3	2	3	1
Psychiatric symptoms	3	1	3	2
Consciousness	3	0	1	0
Language problem	3	1	3	0
Dyskinesia/Dystonia	3	1	3	0
Gait instability and ataxia	1	2	3	3
Brainstem dysfunctions	1	0	1	2
Weakness	2	1	2	0
CASE score	22	8	19	8

## Discussion

This patient with anti-LGI1 encephalitis showed a favorable response to the first-line immunotherapy with a combination of IVMP and PE; however, psychobehavioral symptoms relapsed one month after the initial treatment and five months from the onset. The relapsing symptoms included somnolence, restlessness, and delusions without seizures, some of which resembled her initial manifestations. The decision-making of relapse was challenging in the context of some improvement shown in the antibody profiles, such as negative results of LGI1 antibodies in CSF with fixed CBA, the absence of apparent neuropil reactivity with IIF-TBA, and the absence of recurrence of increased T2/FLAIR signal in the medial temporal lobes.

Patients with anti-LGI1 encephalitis usually respond well to one of the first-line immunotherapies, including IVMP, plasma exchange, or intravenous immunoglobulins (IVIgs). It is reported that 50% improved at least 1 point in the Cognitive Performance Score (CPS) after initial treatment [4]. A multi-center study in China reported that all 109 patients treated with first-line immunotherapy showed improvement in memory, mental ability, and behavioral symptoms, with the modified Rankin Scale (mRS) score decreasing from 3.19 ± 0.13 (range = 1-5) to 1.71 ± 0.09 (range = 1-3) [5].

Despite the favorable response to initial treatment, relapse has been reported in 16.2% [5], 27% [4], and 35% [3] in different populations of anti-LGI1 encephalitis. One study reported that relapse occurred a median of five months after the disease onset [5]. Another study reported relapse in nine of 13 patients during the first six months of the disease (1.7-6 months) [4]. There is no consensus criterion on what constitutes a relapse in patients with autoimmune encephalitis (AE), including anti-LGI1 encephalitis. Some researchers mentioned that the reappearance of symptoms, or aggravation of the original symptoms with an increased Rankin score of 1 point or more, was considered a relapse [5]. However, in a large AE patient cohort, the CASE scores of the 28 patients with a Rankin score of 5 ranged from 11 to 24 [6]. Namely, an AE patient within such a CASE score range remains at the same Rankin score, even if there was an apparent clinical deterioration, thus escaping the definition of relapse. In such cases, the CASE score might be potentially useful to depict subtle worsening, especially in severe patients.

Making a decision on relapse in patients with anti-LGI1 encephalitis may be complicated because relapses are not always accompanied by clinical or laboratory signatures that are pathognomic to anti-LGI1 encephalitis, such as FBDS, increased T2/FLAIR signals in the medial temporal lobes, or an increase in LGI1 antibody titers. Acute brain MRI changes were seen in only three of 19 relapsed patients in one cohort, indicating that clinical relapses are not always synchronized with radiological signatures [5]. In another study [4], LGI1 antibodies became negative with a median follow-up of 20 months in 12 of 16 patients, whose follow-up samples were available; however, the remaining four were still positive even after 20 months of follow-up, although three of them had a good clinical outcome. In another study [5], 16 out of 19 patients with relapse showed positivity for LGI1 antibodies in both serum and CSF, while the remaining three showed positivity for LGI1 antibodies only in serum, as seen in this case. These results imply that positive serum LGI1 antibodies alone may not be interpreted as having high disease activity.

The CASE score was originally developed for rating the disease severity of AE [6]. Nine key items consist of seizure, memory dysfunction, psychiatric symptoms, consciousness, language problems, dyskinesia and dystonia, gait instability and ataxia, brainstem dysfunction, and weakness. Of these, four items are assigned to better capture cognitive components in AE, compared to mRS [7]. It was reported that, in patients with autoimmune encephalitis, a higher CASE score may indicate a higher likelihood of relapse [8]. In addition, this score can quantify subtle changes in severity in patients with substantial cognitive sequelae after the first therapeutic interventions, as shown in our case.

In our case, the CASE score was 22 at presentation (day one) and then improved to 8 after the first immunotherapy (day 34). When psychobehavioral symptoms relapsed, the score was increased to 19 (day 78). When the clinical relapse was being suspected, she had no laboratory evidence supporting relapse of encephalitis, such as hyponatremia, MRI abnormalities, LGI1 antibodies in CSF, or neuropil reactivity on IIF-TBA. Only an increase in the CASE score supported the relapse of encephalitis. Subsequent amelioration of her symptoms after receiving additional immunotherapy supports the relapse of encephalitis in our case.

This study has limitations; (1) this is a retrospective case report, and (2) the LGI1 antibodies were measured with both commercial and established in-house assay at Dalmau’s Lab at the first admission but not at relapse with in-house CBA and IHC at Dalmau’s Lab, the latter in-house assay is more reliable compared with commercial assay, as recently reported [9,10]. Thus, the possibility of false negativity in CSF cannot be excluded. (3) No other biomarkers, such as serum neurofilament light chain [11] or pro-inflammatory cytokines or chemokines, were examined.

Despite this, the CASE score was useful in making a clinical judgment of relapse in our patient. Moreover, using the CASE score as a common language enables evaluation by different physicians during the long course of the disease, which can persist for years. Poorer availability and possible delay in testing of anti-LGI1 antibody, together with possible false positives/negatives of anti-LGI1 antibody in a setting of relapse, can result in either a delayed or an incorrect diagnosis [12], especially in patients with subtle antibody footprints.

## Conclusions

Paraclinical findings, such as brain MRI findings or LGI1 antibody titers in CSF or serum, are generally considered diagnostic biomarkers in anti-LGI1 encephalitis. We may be tempted to reapply them as a monitoring biomarker in case of a possible relapse. However, relying too much on such biomarkers in making a decision of relapse (or non-relapse) can be precarious because of the frequent false positives or false negatives regarding such biomarkers. We believe that the decision on relapse of anti-LGI1 encephalitis must be carefully made based not only on radiological or serological biomarkers but also on neurological assessment of disease-specific symptoms, such as FBDS or a substantial rise in the CASE score.
